# Management of two dogs with post‐operative new‐onset persistent atrial fibrillation following mitral valve repair

**DOI:** 10.1002/vms3.70029

**Published:** 2025-01-15

**Authors:** Kentaro Kurogochi, Masako Nishiyama, Hiroyasu Masaki, Midori Goto Asakawa, Masami Uechi

**Affiliations:** ^1^ JASMINE Veterinary Cardiovascular Medical Center Yokohama Japan; ^2^ Department of Clinical Sciences College of Veterinary Medicine, North Carolina State University Raleigh North Carolina USA; ^3^ You Animal Medical Center Gotemba Japan; ^4^ Veterinary Specialists Emergency Center Kawaguchi Japan

**Keywords:** Holter electrocardiograph, myxomatous mitral valve disease, post‐operative new‐onset atrial fibrillation, rate‐control, rhythm‐control

## Abstract

Post‐operative new‐onset atrial fibrillation (POAF) is a possible complication following cardiac surgery. Digoxin is a drug with positive inotropic and negative chronotropic effects and is listed among antiarrhythmic drugs that can be prescribed in dogs with atrial fibrillation. This report aims at describing the use of digoxin in two dogs with persistent POAF after mitral valve repair.

Case 1 was a Toy Poodle, neutered male, 12 years old, weighing 5.0 kg. The patient was diagnosed with the American College of Veterinary Internal Medicine (ACVIM) Stage D myxomatous mitral valve disease without arrhythmia. The day of the surgery was defined as Day 1. Persistent POAF was diagnosed on Day 30 (1‐min mean heart rate of 206 bpm by in‐clinic electrocardiogram), and treatment with digoxin was started at a dose of 0.0040 mg/kg, p12h. The resolution of atrial fibrillation (AF) was confirmed at the follow‐up visit on Day 58 (1‐min mean heart rate of 162 bpm by in‐clinic electrocardiogram). Case 2 was a mixed‐breed dog, neutered male, 12 years old, weighing 7.6 kg. The patient was diagnosed with ACVIM Stage B2 myxomatous mitral valve disease without arrhythmia. After surgery, the patient had a normal sinus rhythm, but a paroxysmal ectopic atrial rhythm was observed on Day 32. Persistent POAF was diagnosed on Day 130 (1‐min mean heart rate of 216 bpm by in‐clinic electrocardiogram), and treatment with digoxin was started at a dose of 0.0037 mg/kg, p12h. The resolution of AF was confirmed on Day 151 (1‐min mean heart rate of 107 bpm by in‐clinic electrocardiogram).

In this report, the authors suspected that digoxin therapy could have contributed, among other factors, to cardioversion. However, specifically designed studies are needed to confirm this preliminary hypothesis.

## Graphical abstract

Digoxin therapy might restore sinus rhythm in two dogs with persistent post‐operative new‐onset atrial fibrillation after mitral valve repair. Case 1, a 12‐year‐old Toy Poodle (5.0 kg), presented atrial fibrillation on Day 5 and returned to sinus rhythm on Day 58 after surgery. Case 2, a 12‐year‐old mixed breed (7.6 kg), presented atrial fibrillation on Day 130 and achieved sinus rhythm on Day 151 post‐surgery.

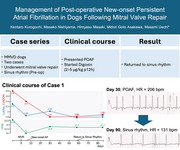

## 1 BACKGROUND

Mitral valve repair (MVR) is a curative treatment option for developed myxomatous mitral valve disease (MMVD) (Kanemoto et al., 2021; Masashi & Masami, 2020). Dogs that undergo MVR may develop post‐operative new‐onset atrial fibrillation (POAF). From the authors’ experience, many of them return to sinus rhythm during the early post‐operative period and rarely persist, which aligns with the reports within the field of human medicine (Dobrev et al., 2019). Nonetheless, we experienced two cases of persistent POAF in dogs, which we defined, based on the human literature (January et al., 2014), as a newly occurring POAF lasting for more than 1 week. It should be noted that this definition is not universally accepted in the present veterinary practice, as it is often challenging to determine the exact onset of atrial fibrillation in dogs.

The diagnosis of atrial fibrillation primarily relies on the combination of clinical and electrocardiographic findings. Common clinical signs include lethargy, weakness, exercise intolerance, coughing, and dyspnea (Menaut et al., 2005). Electrocardiogram (ECG) findings include an absence of discernible P waves and an irregular ventricular response due to chaotic and disorganized atrial electrical activity. Fibrillatory f waves of varying amplitude (undulating baseline) might be identified on the ECG trace (Willis et al., 2018).

For controlling atrial fibrillation caused by mitral valve disease and dilated cardiomyopathy, the ventricular response is usually managed by diltiazem and digoxin (Pedro et al., 2020), preferentially in combination (Jung et al., 2016), amiodarone (Pedro et al., 2012; Romito et al., 2024; Saunders et al., 2006), and, less commonly, beta‐blockers (Borgeat et al., 2021). Successful rate‐control is crucial for improving the prognosis of dogs with cardiac disease. In a report focusing on dogs with mitral valve disease and dilated cardiomyopathy accompanied by atrial fibrillation, higher heart rate (HR) during echocardiogram represented independent predictors of negative outcome (Romito et al., 2023). Also, a HR less than 160 bpm, measured by a standard ECG in a clinical setting, was associated with more prolonged survival (Jung et al., 2016). However, it is essential to note that the interpretation of HR based on in‐clinic ECGs may not accurately reflect the mean HR in the dog's home environment. Gelzer et al. (2015) reported that in‐clinic HR overestimated the mean HR of a Holter recording by 26 bpm. Recent research has demonstrated that dogs with atrial fibrillation and a 24‐h mean HR of less than 125 bpm, as captured on a Holter ECG, have significantly extended survival times (Pedro et al., 2018, 2023). Therefore, achieving a mean HR of less than 125 bpm seems to be a practical target for rate‐control therapy. However, all these studies focus on naturally occurring atrial fibrillation accompanied by underlying heart disease, and there is no report on POAF following cardiac surgery in veterinary practice.

Digoxin has both positive inotropic and negative chronotropic effects, making it a potential treatment agent for supraventricular arrhythmias in dogs with heart disease. mainly when used at low, safer doses. Traditionally, a common approach is to prescribe digoxin at a standard dose (from 0.0020 to 0.0050 mg/kg every 12 h) and to check digoxin serum concentration (8–10 h from administration approximately after 1 week from prescription) to ensure that it is within the ideal range (0.6–2.0 ng/dL) and reduce the risks of digoxin toxicity (Gelzer et al., 2009; Pedro et al., 2018, 2020).

This report presents the first cases of persistent POAF in two dogs that occurred after MVR and returned to sinus rhythm.

## 2 CASE PRESENTATION

**FIGURE 1:**
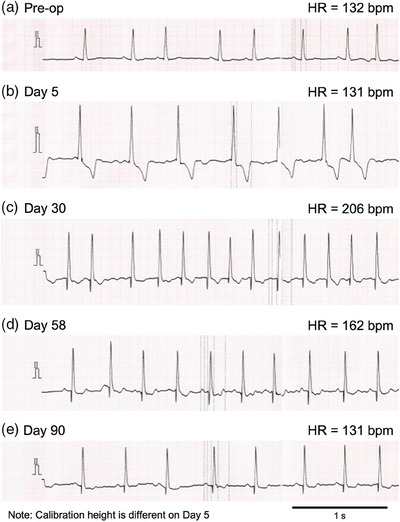
Electrocardiographic findings in Case 1. Before the surgery, only occasional premature atrial contractions were observed. Atrial fibrillation occurred immediately after the surgery and persisted until Day 30. On Day 58, sinus rhythm was achieved after starting digoxin administration and was maintained without recurrence of atrial fibrillation, although occasional premature atrial contractions were still observed. Paper speed: 50 mm/s, HR, heart rate; bpm, beats per minute.

**FIGURE 2:**
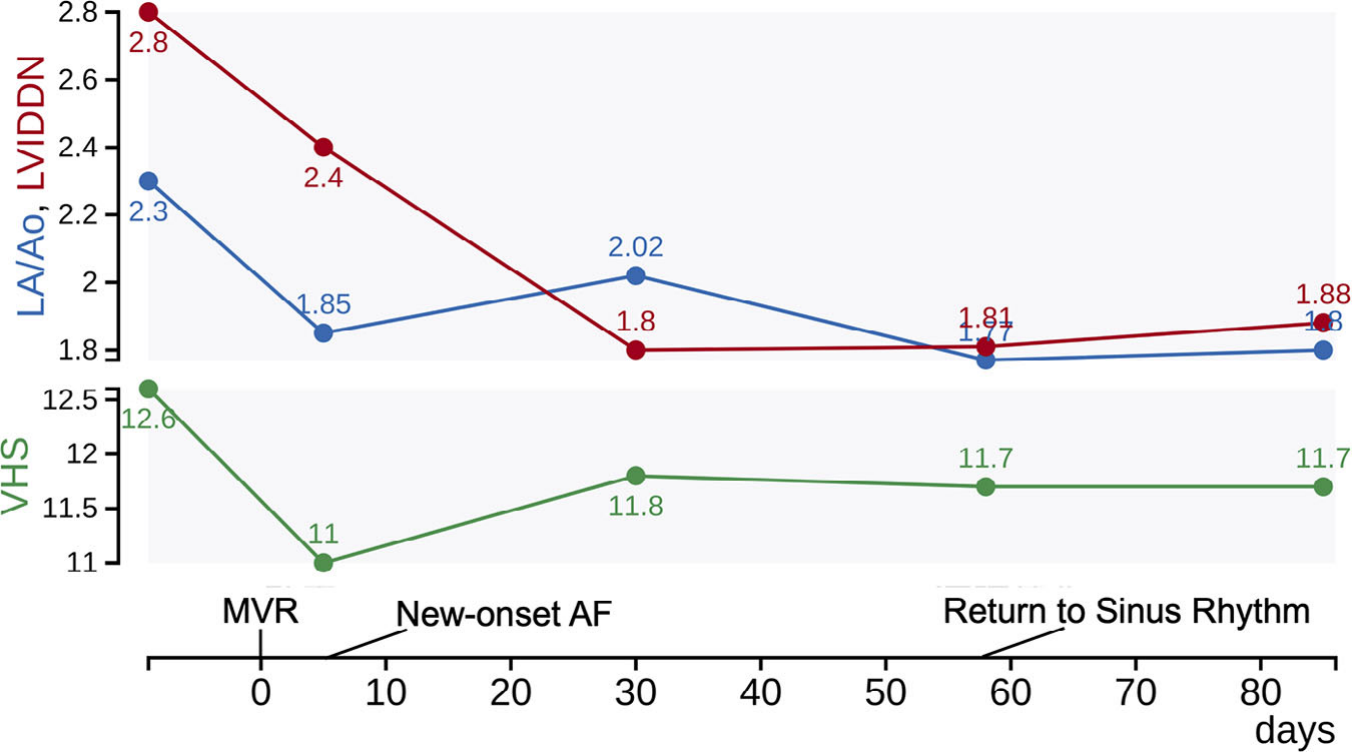
Case 1: clinical course of atrial fibrillation onset to sinus rhythm restoration. AF, atrial fibrillation; LA/Ao, left atrial to aortic ratio; LVIDDN, left ventricular end‐diastolic internal diameter normalized to body weight; MVR, mitral valve repair; VHS, vertebral heart size.

Case 1 was a 12‐year‐old neutered male Toy Poodle weighing 5.0 kg. Treatment for frequently repeated pulmonary edema due to MMVD was received at the referring animal hospital. Prescriptions at that time were pimobendan (0.53 mg/kg p8h), torsemide (0.34 mg/kg p12h), and spironolactone (1.3 mg/kg p12h). The patient was referred to our veterinary centre to consider surgical treatment. In the thoracic radiogram, the vertebral heart size (VHS) was 12.6, and the vertebral left atrium size was 3.2. Echocardiographic findings were left atrial to the aortic ratio (LA/Ao) 2.3, left ventricular end‐diastolic internal diameter normalized to body weight (LVIDDN) 2.8, fractional shortening 40%, left ventricular early diastolic inflow velocity 1.3 m/s, and tricuspid regurgitant velocity 2.8 m/s. Due to persistent pulmonary edema, the patient had been hospitalized in the intensive care unit for 3 weeks before the day of referral. Based on these medical backgrounds, the disease severity was diagnosed as Stage D MMVD based on the American College of Veterinary Internal Medicine (ACVIM) consensus statement (Keene et al., 2019). A preoperative electrocardiography (ECG) revealed a single atrial premature contraction (Figure 1a). The surgical intervention, MVR, was performed because the prognosis of medical treatment seemed to be limited. Pre‐ and post‐operative heart size over time (VHS, LA/Ao, and LVIDDN) are shown in Figure 2. Post‐operative prescription was pimobendan (0.36 mg/kg p12h), furosemide (0.9 mg/kg p12h), and temocapril (0.1 mg/kg p12h). On Day 5 (during post‐operative hospitalization), POAF occurred (Figure 1b) but did not accompany significant tachycardia (1‐min mean HR of 130 bpm by in‐clinic ECG, range 76–128 bpm by physical examination). On Day 30, POAF was still observed by standard ECG, with tachycardia (1‐min mean HR of 206 bpm by in‐clinic ECG; Figure 1c), then we considered it persistent POAF, and a Holter ECG was performed on Day 30–32 revealing a 24‐h mean HR of 140 bpm. The electrolyte levels at the time of diagnosis were as follows: sodium 148 mEq/L, potassium 3.6 mEq/L, chloride 118 mEq/L, and magnesium 1.9 mg/dL. As an introduction to rate control, digoxin was started at a dose of 0.0040 mg/kg p12h from Day 33, and the blood digoxin concentration on Day 43 was 0.9 ng/mL. The patient unexpectedly returned to sinus rhythm on Day 58 (1‐min mean HR of 162 bpm by in‐clinic ECG; Figure 1d). On Day 90, the LA/Ao was 1.8, with no recurrence of atrial fibrillation (Figure 1e). The patient is confirmed to be alive without onset of heart failure or recurrence of atrial fibrillation at Day 1139.

**FIGURE 3:**
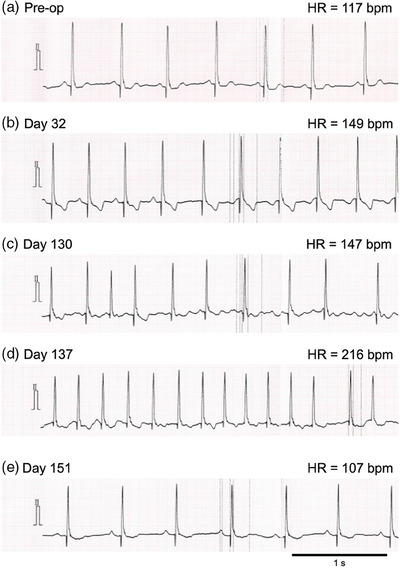
Electrocardiographic findings in Case 2. Prior to the surgery, the patient was in sinus rhythm. On Day 32, the paroxysmal ectopic atrial rhythm was detected, and on Day 130, the transition to atrial fibrillation was confirmed. Despite intensified treatment for heart disease, no improvement in arrhythmia was observed on Day 137, and the patient was diagnosed with persistent atrial fibrillation. After starting digoxin administration, the patient returned to sinus rhythm on Day 151. Paper speed: 50 mm/s, HR, heart rate; bpm, beats per minute.

**FIGURE 4:**
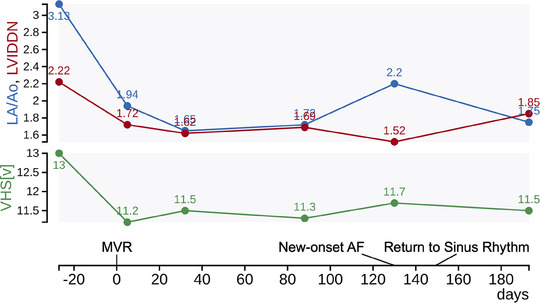
Case 2: clinical course of atrial fibrillation onset to sinus rhythm restoration. AF, atrial fibrillation; LA/Ao, left atrial to aortic ratio; LVIDDN, left ventricular end‐diastolic internal diameter normalized to body weight; MVR, mitral valve repair; VHS, vertebral heart size.

**FIGURE 5:**
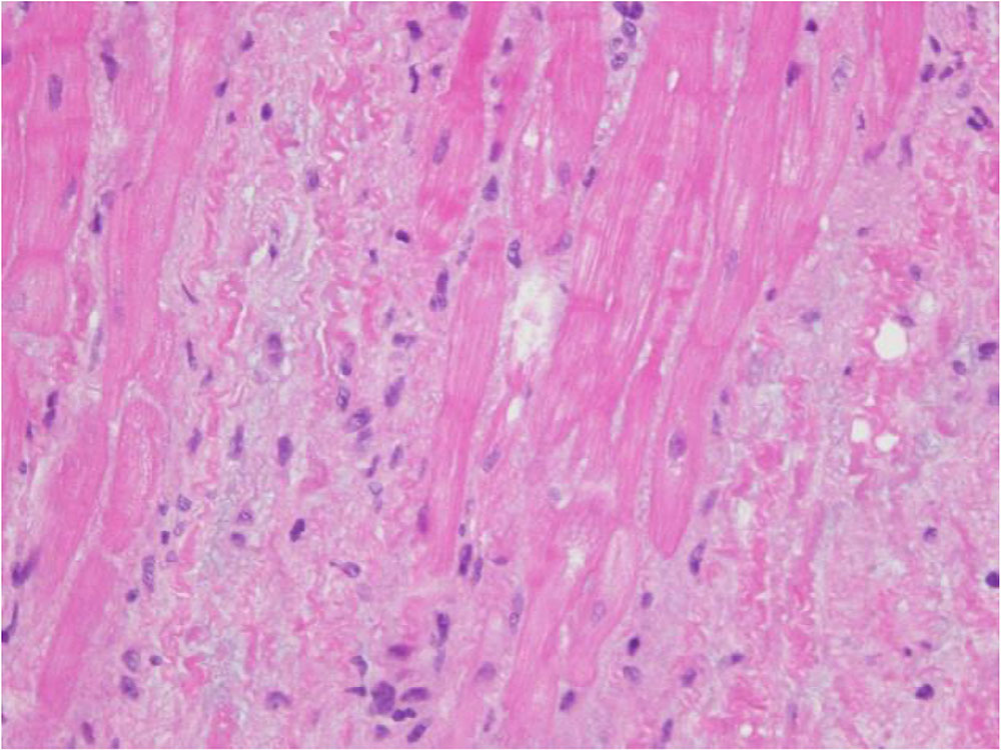
Pathological findings of the biopsied left atrium in Case 2. In the myocardial cells, myocardial atrophy and degeneration with hypereosinophilic sarcoplasm and hyperchromatic condensed nuclei were observed, along with contraction bands. Multifocal myocardial fibrosis and mild interstitial infiltration of mononuclear cells were present. Hematoxylin and Eosin.

Case 2 was a 12‐year‐old neutered male mixed‐breed dog weighing 7.6 kg. The referring animal hospital prescribed pimobendan (0.40 mg/kg p8h), torsemide (0.13 mg/kg p12h), and furosemide (0.64 mg/kg p12h) because of the presence of a heart murmur and findings of cardiac enlargement. The patient was referred to our centre to consider surgical treatment. In the thoracic radiogram, VHS was 13.0, and vertebral left atrium size was 3.8. Findings from the echocardiogram were LA/Ao 3.1, LVIDDN 2.2, fractional shortening 57%, left ventricular early diastolic inflow velocity 1.3 m/s, and tricuspid regurgitant velocity 3.0 m/s. The patient was diagnosed with ACVIM Stage B2 MMVD. Despite no history of congestive heart failure, the prognosis was considered poor due to significant cardiac dilation, making MVR a viable treatment option. Preoperative diuretics were continued due to continuous and marked worsening cardiac enlargement observed during periodic examinations at the referring hospital (data not shown), and concerns that withdrawing these medications might cause pulmonary edema before the surgery date. It should be noted, however, that these treatments are not standard medical treatments for Stage B2 dogs (Keene et al., 2019). A preoperative electrocardiography showed sinus rhythm (Figure 3a). The patient underwent MVR (Day 1); the course of heart size is shown in Figure 4. The patient underwent a consensual left atrial biopsy (after obtaining written consent from owners). This procedure was not performed as strictly related to the clinical management of the dogs, but as a part of a research project conducted in the same period of MVR. Histopathology of the biopsied left atrium revealed multifocal, moderate myocardial fibrosis, mild interstitial mononuclear infiltrates, myocardial atrophy, and degeneration, characterized by fragmented and hypereosinophilic sarcoplasm, and occasional contraction bands (Figure 5). Post‐operative prescription was pimobendan (0.33 mg/kg p12h) and furosemide (0.64 mg/kg p12h) to control post‐operative heart condition. On Day 5, sinus rhythm was recorded in standard ECG (Figure 3b). On Day 32, the paroxysmal ectopic atrial rhythm was revealed (Figure 3c). On Day 130, POAF was observed by standard ECG (1‐min mean HR of 147 bpm by in‐clinic ECG) (Figure 3d). At that time, pimobendan was continued, furosemide was switched to torsemide (0.12 mg/kg p12h), and benazepril (0.30 mg/kg p24h) was prescribed. All these therapeutic changes were intended to manage potential triggers of acute atrial fibrillation (such as excessive left atrial volume overload), not on the base of canine literature, but rather on the base of the human one (Chyou et al., 2023). However, tachycardia (1‐min mean HR of 216 bpm by in‐clinic ECG) was present on Day 137. Then, a Holter ECG was performed (Days 137−139), and persistent POAF was diagnosed (24‐h mean HR of 134 bpm). The electrolyte levels at the time of diagnosis were as follows: sodium 149 mEq/L, potassium 3.3 mEq/L, chloride 106 mEq/L, and magnesium 1.5 mg/dL. As an introduction to rate control, digoxin was initiated at a dose of 0.0037 mg/kg p12h on Day 140. By Day 151, the blood digoxin concentration was 0.6 ng/mL, and sinus rhythm was restored (1‐min mean HR of 107 bpm by in‐clinic ECG; Figure 3e). On Day 220, the LA/Ao was 1.7, and up to Day 783, there was no recurrence of atrial fibrillation nor any signs of heart failure. Nevertheless, a recurrence of atrial fibrillation was identified during a routine post‐operative checkup on Day 931, accompanied by enlargement of the left atrium (LA/Ao of 1.98). The in‐clinic 1‐min mean HR remained steady at 150 bpm. Evaluation of the HR by Holter monitoring and a review of the treatment were considered. However, since the patient's in‐hospital HR was below 160 bpm (Jung et al., 2016) and the owner did not request further examination, it was decided to continue the current treatment until Day 973, when this report was written.

## 3 DISCUSSION AND CONCLUSIONS

In human medicine, POAF is a common complication following cardiac surgery (Dobrev et al., 2019; Greenberg et al., 2017; LaPar et al., 2014), and it is also a possible arrhythmia in dogs after MVR in our institution, but its information is limited in veterinary medicine. The presence of atrial fibrillation is a significant concern for veterinarians due to its relevance to morbidity and mortality (Jung et al., 2016; Pedro et al., 2018). A priority in the management of POAF is the identification and treatment of potential triggers because rate‐ and rhythm‐control may be less likely to succeed until the acute illness improves in human medicine (Chyou et al., 2023). In this report, we identified heart enlargement (especially of the left atrium) at the time of diagnosis with POAF. This is why we used pimobendan, ACE inhibitors, and diuretics at this time to reduce the left atrial pressure (Ishikawa et al., 2010; Suzuki, Fukushima, et al., 2011, Suzuki, Ishikawa, et al., 2011). However, it should be noted that these treatment strategies have not been prescribed exactly in agreement with recommendations from the current ACVIM guidelines on canine MMVD (Keene et al., 2019).

The pathophysiology of spontaneous atrial fibrillation in dogs with naturally acquired heart disease is multifactorial and only partially understood. Nonetheless, several risk factors have been considered, including breed, age, body weight, presence of congestive heart failure, left and right atrial enlargement, pre‐existing cardiac disease, and left atrial pressure (Arcuri et al., 2024; Borgarelli et al., 2004).

Differently from spontaneous atrial fibrillation, the POAF can be caused by tissue inflammation of the myocardium and scarring at the atrial incision site (Chyou et al., 2023; Pathak et al., 2013). On a microscopic level, changes observed in the atrial myocardium include cellular hypertrophy, myolysis, dedifferentiation, fibrosis, and apoptosis, along with disruption of the mitochondria and sarcoplasmic reticulum (Pathak et al., 2013). In the present report, surgical‐related myocardial inflammation and atrial scarring likely played a relevant role in the development of POAF. Additionally, it could be hypothesized that the advanced clinical stage in Case 1 (the ACVIM Stage D) and the progression of the heart condition in Case 2 (with histological evidence of myocardial fibrosis and atrophy of the left atrium) might have represented additional contributing factors in the development of POAF.

In humans, the management of POAF involves also controlling the underlying substrates/triggers, such as atrial scars and volume/pressure overload (Chyou et al., 2023; Kotecha & Castellá, 2020). Although the rationale use for post‐operative ACE inhibitor is unclear, discontinuation of the drug may increase the risk of developing POAF in humans (Mathew et al., 2004). This justifies part of our medical choices; indeed, both dogs from this report were under ACE inhibitors after surgery. Concerning additional oral drugs aimed at controlling MMVD and left atrial pressure, in line with veterinary literature, they included pimobendan and loop diuretics (Keene et al., 2019; Wess et al., 2020). Therefore, these therapies were intensified prior to arrhythmia treatment in the present report. In persistent atrial fibrillation, a report on 16 dogs suggested that the achievement of sinus rhythm following MVR may potentially affect long‐term prognosis (Kazuki & Masami, 2022). Therefore, it seems clinically significant to obtain sinus rhythm (rhythm control) also thanks to the use of antiarrhythmic drugs. Interestingly, the two cases in this report received digoxin among cardiac medications, and both had a good clinical course.

Digoxin, a cardiac glycoside, is utilized in the management of conditions such as atrial fibrillation and heart failure. It enhances the contractile force of the heart muscles and moderates the HR by influencing the sodium‐potassium ATPase pump. Though it appears to have only modest electrophysiologic effects (Sarter & Marchlinski, 1992), the vagotonic effect of digoxin is considered to play a predominant role in the drug's ability to control HR in patients with atrial fibrillation (Rosen et al., 1975). The direct myocardial effect increases the refractory period and slows conduction in atrial tissue. This increase in conduction time and refractory period would tend to provide protection against atrial fibrillation (Hoffman, 1990). A stimulating central effect on the sympathetic tone (Stark et al., 1972) and the parasympathetic tone (Gillis, 1975) has also been noted in experimental settings, with the latter being responsible for chronotropic and dromotropic effects. Improvement in the neurohormonal profile of heart failure patients has been achieved in human medicine, possibly related to digoxin modulating effects on various neurohormonal abnormalities, including improvement on baroreceptor function, a vagomimetic effect, a sympathoinhibitory effect, a decrease in circulating neurohormones with dose‐related effects on neurohormones, and antifibrotic effects (Gheorghiade et al., 2004). Under digoxin therapy, substantial increase in parasympathetic activity was observed in humans, whereas low‐frequency power, an index of baroreflex activity, was also significantly increased, indicating that digoxin acts to ameliorate the autonomic dysfunction of patients with heart failure (Krum et al., 1995). However, in dogs from the present report, it is impossible to conclusively establish if digoxin contributed, at least in part, to cardioversion or if the interruption of atrial fibrillation occurred uniquely as a consequence of progressive attenuation of surgery‐related inflammation.

This report is a retrospective case series and, therefore, has several limitations, including lack of controls, absence of continuous Holter monitoring, and treatments that were not standardized having been administered by several different clinicians. Furthermore, while this case series demonstrates a unique clinical course where sinus rhythm was achieved after the administration of digoxin, it should be considered that the use of amiodarone (Pedro et al., 2012) or electrical cardioversion (Bright & zumBrunnen, 2008) can be a suitable choice for achieving rhythm control.

In conclusion, the restoration of sinus rhythm in the two cases of persistent POAF was unexpectedly achieved by using digoxin for two dogs. The authors believe that digoxin therapy, among other factors, could have contributed to cardioversion. However, given the extraordinary post‐operative situation, further specifically designed studies are needed to confirm this preliminary hypothesis.

## AUTHOR CONTRIBUTIONS

Kentaro Kurogochi contributed to the preparation of materials, data collection, and wrote the first draft of the manuscript. Masako Nishiyama and Hiroyasu Masaki contributed to data collection and case management. Midori Goto Asakawa contributed to pathological investigations. Masako Nishiyama and Masami Uechi provided critical feedback and supervision in the interpretation of the research. All authors have read and approved the final version of the manuscript.

## CONFLICT OF INTEREST STATEMENT

The authors declare no conflicts of interest.

## FUNDING INFORMATION

This research did not receive any specific grant from funding agencies in the public, commercial, or not‐for‐profit sectors.

## CONSENT STATEMENT

Data were collected from patients following the written informed consent from the owners for publication.

### ETHICS STATEMENT

Patients' data were provided following consent from the owners, and the data were securely stored. The entire study process was approved by the ethical committee of JASMINE Veterinary Cardiovascular Medical Center (approval number: 230829‐3) in accordance with the Declaration of Helsinki and the guidelines of the Council for International Organizations of Medical Sciences.

### PEER REVIEW

The peer review history for this article is available at https://publons.com/publon/10.1002/vms3.70029.

## Data Availability

The data in this study are available from the corresponding author upon reasonable request.
